# Clustering pattern and evolution characteristic of microRNAs in grass carp (*Ctenopharyngodon idella*)

**DOI:** 10.1186/s12864-023-09159-x

**Published:** 2023-02-13

**Authors:** Huiqin Niu, Yifan Pang, Lingli Xie, Qiaozhen Yu, Yubang Shen, Jiale Li, Xiaoyan Xu

**Affiliations:** 1 Key Laboratory of Freshwater Aquatic Genetic Resources Ministry of Agriculture and Rural Affairs, Shanghai Ocean University, Shanghai, China; 2 National Demonstration Center for Experimental Fisheries Science Education, Shanghai Ocean University, Shanghai, China; 3 Shanghai Engineering Research Center of Aquaculture, Shanghai Ocean University, Shanghai, China

**Keywords:** microRNA, miRNA clusters, Grass carp, Evolution, Purifying selection

## Abstract

**Background:**

A considerable fraction of microRNAs (miRNAs) are highly conserved, and certain miRNAs correspond to genomic clusters. The clustering of miRNAs can be advantageous, possibly by allowing coordinated expression. However, little is known about the evolutionary forces responsible for the loss and acquisition of miRNA and miRNA clusters.

**Results:**

The results demonstrated that several novel miRNAs arose throughout grass carp evolution. Duplication and de novo production were critical strategies for miRNA cluster formation. Duplicates accounted for a smaller fraction of the expansion in the grass carp miRNA than de novo creation. Clustered miRNAs are more conserved and change slower, whereas unique miRNAs usually have high evolution rates and low expression levels. The expression level of miRNA expression in clusters is strongly correlated.

**Conclusions:**

This study examines the genomic distribution, evolutionary background, and expression regulation of grass carp miRNAs. Our findings provide novel insights into the genesis and development of miRNA clusters in teleost.

**Supplementary Information:**

The online version contains supplementary material available at 10.1186/s12864-023-09159-x.

## Introduction

According to large-scale studies of microRNAs (miRNAs) in various species, miRNAs are widely dispersed, and many of them tend to be clustered together on the same chromosome in animals [1]. Based on location distribution and sequence similarity, miRNAs can be divided into three categories: miRNA clusters, families, and single miRNAs. A cluster is defined if two or more miRNAs share a close physical distance (less than 10 kb), whereas a miRNA family is defined if two or more miRNAs sequence share a higher similarity. A miRNA family may be part of one or more clusters, and a cluster may contain one or more families [2]. Typically, expansion of miRNA families occur via tandem or segmental duplications [3]. The development of new miRNA families appears to be an ongoing process, resulting in the emergence of numerous very young and even species-specific miRNAs [4]. Despite the rapid expansion of our understanding of the development of miRNAs, very little is known about the evolutionary characteristics and patterns of miRNA clusters in vertebrates.

The origin of miRNAs can be traced back to early metazoans. Evolutionary forces such as duplication, inversion, mutation, amplification, and other forms of genetic drift that shape the genome may be the most influential factors in the origin and evolution of miRNA genes [5]. During long-term evolution, the repertoire of miRNAs in animals gradually expanded [6]. Although miRNAs are highly conserved noncoding RNA molecules, numerous novel and lineage-specific miRNAs [7–10] have been discovered in multiple taxa and evolve quickly and undergo a fast turnover [11]. Animal miRNA evolution seems to have been a relatively dynamic process. The dramatic expansion of the miRNA repertoire during evolution appears to have been associated with the emergence of phenotypic variation in closely related species [12, 13]. A birth and death model of miRNA evolution, based on observations in *Drosophila*, explains the vast flux of evolutionarily young miRNAs in multiple lineages [14].

Genome duplication and de novo formation are crucial mechanisms for miRNA cluster formation [15]. Among *Populus* miRNA clusters, 37.2% of miRNAs were derived from duplication events, while de novo formation resulted in 62.8% of *Populus*-specific miRNAs [16]. Yu et al. [17] analyzed the homology among miRNAs in 38 miRNA clusters and found that these clustered miRNAs were formed through local duplication of the same ancestral gene. Genes arranged in clusters may or may not be homologous to each other. According to miRBase [18], > 30% of animal miRNAs are organized into clusters, and some of these clusters produce polycistronic transcripts [19, 20]. Typically, a miRNA cluster contain two or three miRNA genes. However, more miRNA genes have also been identified in one cluster, such as the human mir-302 cluster, which includes eight miRNA genes. Megraw et al. [21] analyzed miRNA clusters in the genomes of four species: human, mouse, rat, and chicken, and found that more than 30% of miRNA genes were clustered when the defined MID (maximum inter-miRNA distance) was 1 kb. In addition, the rice genome contains 18 miRNA clusters containing 52 miRNA genes [22].

Notably, miRNAs belonging to the same cluster indicate the possibility of co-regulation. Experimental evidence demonstrated that clustered miRNA loci form an operon-like gene structure and are transcribed from a shared promoter, resulting in a cooperative function [17] and sustained expression levels [23] for these miRNAs [24]. Co-transcription of the same genomic locus or participation in comparable biological functions partially explain the co-expression of miRNAs in the same module. Clustered miRNAs exhibit combined effects and coordinated regulatory actions [25]. The cooperative and combinatorial targeting capabilities of miRNAs result in precise and robust gene regulation at both the single-gene and gene-network levels [26], for example targeting genes in the same pathway [27]. In recent years, numerous studies have sought to elucidate the expression of miRNA clusters. For example, the miR-302/367 cluster regulates mitophagy and *Pseudomonas aeruginosa* clearance in macrophages by targeting *NF-kB* [28]. In contrast, the miR-191/425 cluster has a crucial effect on breast cancer cell malignancy initiation and progression [29]. The activated expression of miR-166 k-166 h has been demonstrated to increase rice resistance to fungal infections in rice [30]. Thus, miRNA clusters play a crucial role that is more complex and efficient than that of individual miRNAs.

The grass carp (*Ctenopharyngodon idella*), a member of the Cyprinidae family, is one of the most important cultivated species in freshwater aquaculture. It's the highest output for a single species worldwide [31]. Although research on miRNAs is expanding, there is no information on the evolution of grass carp miRNAs. In this study, we expanded the number of grass carp miRNAs by taking advantage of the genomic resources and transcriptome data for *C. idella*. We performed a systematic, genome-wide survey of *C. idella* miRNAs to comprehend the genomic arrangement of miRNAs. We investigated the origin and evolution of miRNAs and determined the selection pressure on miRNAs. The expression profiles of miRNAs in various tissues were compared. The evolution, expression, and genomic data were merged to completely appreciate why miRNA genes were clustered and how this influences miRNAs' evolution, expression, and function.

## Results

### Identification of miRNAs in *C. idella*

**Fig. 1:**
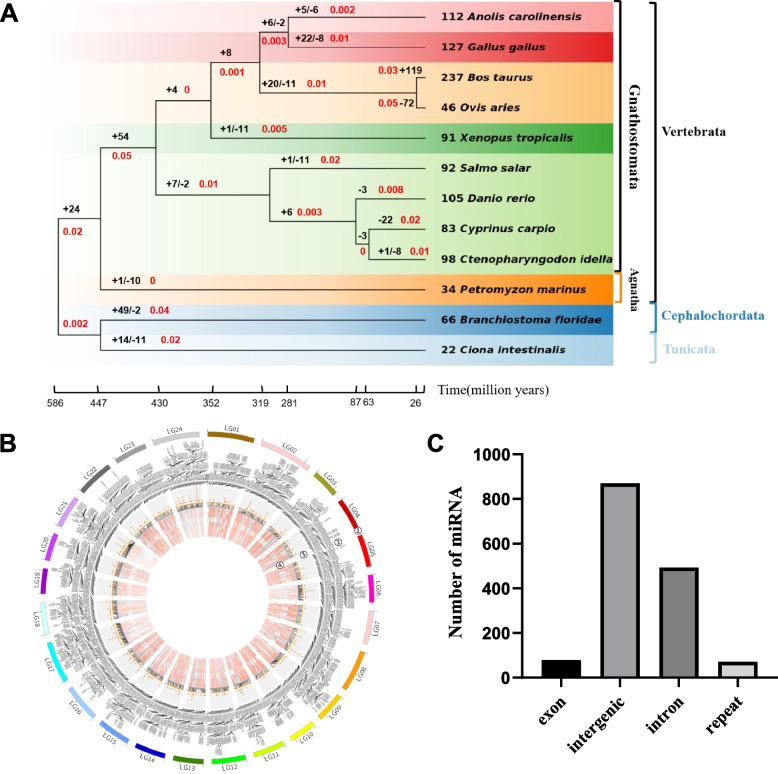
Genomic distribution of grass carp miRNAs and miRNA phylogeny in Chordata. **A** Distribution of miRNA genes in the genome of *C.idella.* Tracks from outside in Track 1: 24 linkage groups of the genome. Track 2: Name of miRNAs (prefix “miR-” was omitted). Track 3: miRNA loci, clustered miRNAs at adjacent positions are displayed in a stacked style. Track 4: miRNA expression (normalized count), from inside out: muscle, intestine, heart, hatching stage, gill, brain. **B** Birth and death rates of miRNA families. Twelve animal species were used in the study, and estimated rates of miRNA families gain and loss, as inferred by parsimony procedure, are shown. Branch lengths reflect evolutionary divergence times per million years inferred from timetrees (http://www.timetree.org/). The number of gained ( +) and lost ( −) families and the rates of miRNA family gain per million years (MY) (in red) are indicated next to each branch. Each species' total number of families is shown before its name. **C** The physical locations of miRNAs in the grass carp genome

To systematically identify miRNAs in *C. idella*, 18 RNA-seq libraries from the brain, muscle, gill, intestine, heart, and hatching stage were sequenced. We identified 1,506 miRNA precursors (pre-miRNAs) that can mature into 1,513 miRNAs (Fig. 1B, Table S1). 1,442 miRNAs are unique to grass carp, arranged into 244 miRNA families containing an average of 1.34 genes per family.

We used the parsimony method to investigate the birth and death rates and ancestor miRNA gene families during animal evolution (Table S2). There is an increase in the number of miRNA families across all studied animal species (from 19 ancestral families to 22–237 families), indicating that only a tiny portion of the miRNA system was directly inherited from the common ancestor during evolution and that a large part of the system diverged. However, across the Vertebrata, Cephalochordata, and Tunicata clades, the rates at which species acquired miRNA families differed (0.001–0.05 families per million years; Fig. 1A). *O.aries* have the highest gain rate of miRNA family. However, *A.carollnensis* showed the lowest. Notably, most terminal branch net gain rates were higher than interior branch net gain rates. The appearance of new miRNA families brings about this process. Our research indicates that miRNA families in animal species have a high turnover rate. Thus, the newly created miRNA families appeared rapidly but were lost over long evolutionary periods.

### Expansion of miRNA clusters by gene duplication

To study the underlying mechanism of emergence of miRNA genes, we investigated miRNA locations in the genome of grass carp, including intergenic area, introns, exons, and repetitive regions. Of these miRNAs, 71 were found within repetitive regions, 79 within exons, 493(32.20%) within introns, and 870 throughout the intergenic area. Consequently, there were 1.76 times more miRNAs in the intergenic area than in the introns of coding genes (Fig. 1C). In contrast, the number of miRNAs in the region containing repetitive sequences was less than a tenth of that found in intergenic areas.

**Fig. 2:**
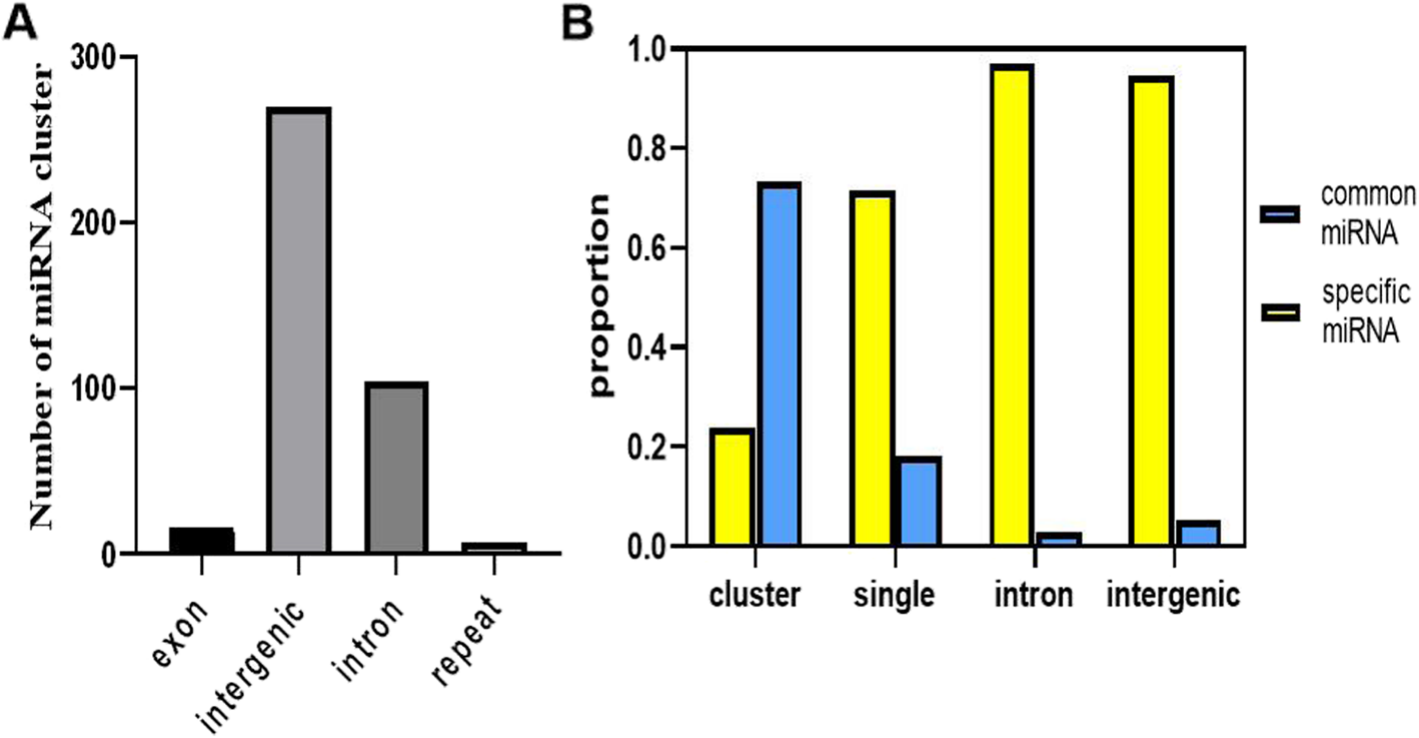
Evolutionarily conserved (common) miRNAs are significantly enriched in clusters. **A** The total number of physical locations of the 397 miRNAs in the miRNA cluster in the grass carp genome. **B** The proportion of common and specific miRNAs in miRNA clusters, single miRNAs, introns, and exons. Conserved (common) miRNAs are enriched in miRNA clusters

We further noticed that the miRNA clusters in grass carp. Our data show that 171 clusters were predicted for 397 miRNA genes, accounting for 26.2% of all miRNAs, 345 (23.93%) of all grass carp-specific miRNAs, and 52 (73.24%) of all common miRNAs (Fig. 2B). Each cluster consists of different numbers of miRNAs (2–7). Of these miRNAs, 7 (1.76%) were discovered in repetitive areas, 16 (4.03%) in exons, 270 (68.01%) throughout the intergenic region, and 104 (26.20%) in introns (Fig. 2A). By randomly permuting the chromosomal sites of miRNAs, we discovered that common miRNAs are considerably enriched in clusters in grass carp. Then, we compared the numbers of miRNA clusters that originated by different mechanisms. The production pattern of 56 miRNAs within the clusters of miRNA genes could not be established. Besides these, 159 are de novo*,* and 52 are inherited (Table S3). Fifty-two from inheritance generated 130 through replication, 104 from cis-duplications, and 26 from trans-duplications (Table S4). Overall, grass carp miRNAs are somewhat clustered, and our findings imply that de novo creation accounts for a more significant proportion of the grass carp miRNA repertoire expansion than duplication.

### Grass carp-specific miRNA evolved at a faster pace

**Fig. 3:**
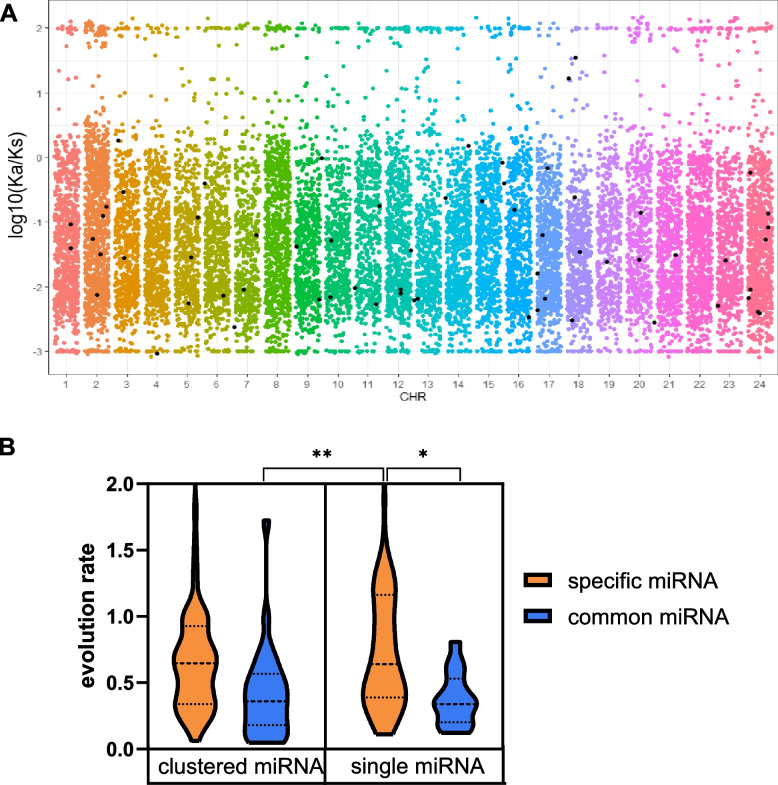
Grass carp-specific miRNA evolved at a faster pace. **A** Ka/Ks ratios for all coding regions of grass carp. **B** Comparison of evolutionary rates between common miRNAs and specific miRNAs. K_p_, K_f_, and K were calculated by pairwise comparison of the orthologous miRNAs between grass carp and zebrafish. Multiple comparisons of samples by means of LSD (least significant difference) were carried out using R. Statistical significance was set at *P* < 0.05. *p < 0.05 and **p < 0.01 indicated statistical significance

The Ka value of miRNA is frequently lower than the Ks value in grass carp (Fig. 3A). The common miRNA evolved at a slower rate than miRNAs specific to grass carp (Fig. 3B, Table S5). Interestingly, the rate of single specific miRNAs was significantly higher than that of clustered common miRNAs. Clustered miRNAs are more conserved and evolve more slowly than grass carp-specific miRNAs, which evolve faster.

**Fig. 4:**
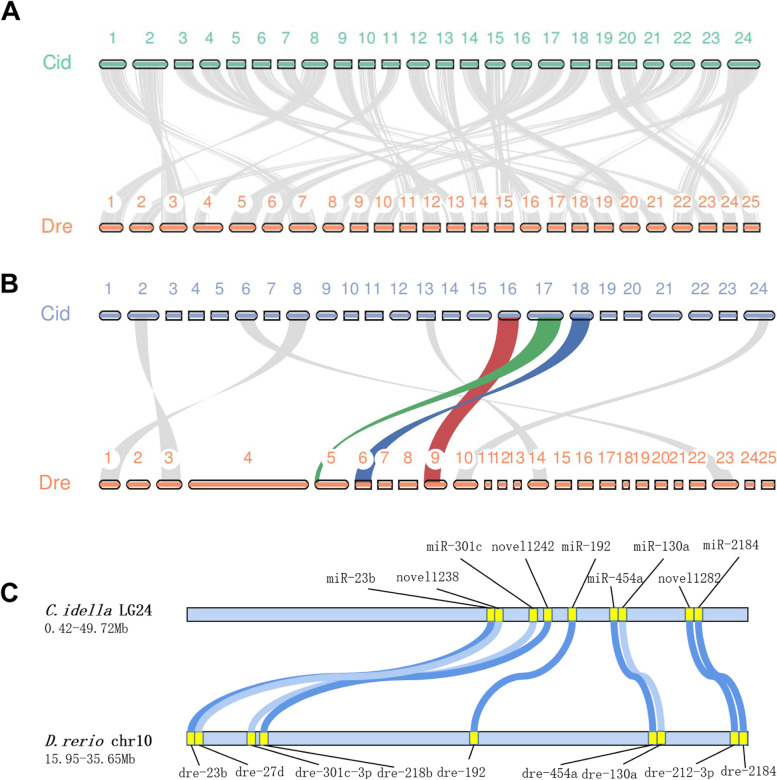
Large degree of variation in non-coding regions. **A** The collinearity analysis of all of the coding regions in grass carp. Proteins are more conservative. **B** The collinearity analysis of all of the miRNAs in grass carp. **C** Detailed information of miRNAs collinear blocks at chromosome 24. Collinear blocks between the two species are shown in blue

A protein-based collinearity analysis revealed that the order of genes in grass carp and zebrafish are remarkably conserved (Fig. 4A, Table S6). However, just a few miRNA block sites were shared by grass carp and zebrafish (Fig. 4B, Table S7). 9 miRNA collinear blocks with 64 miRNAs were discovered, with 40 (62.5% of the total) in clusters (Fig. 4C and Fig. S1). This study found that protein-coding regions were highly conserved even among closely related species, but non-coding genes differed significantly.

### Expression pattern of grass carp-specific miRNAs

**Fig. 5:**
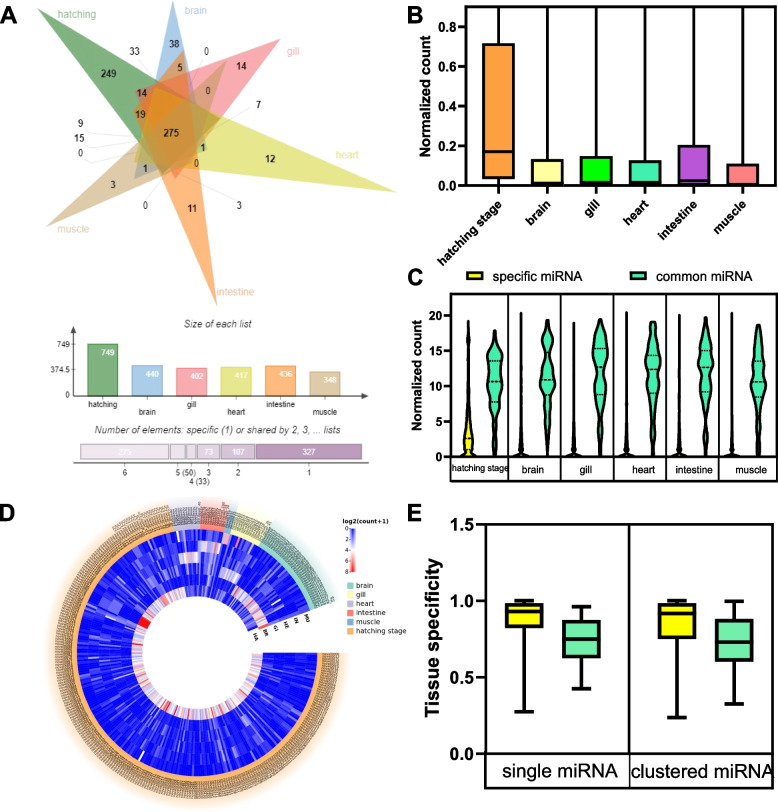
Expression pattern of grass carp-specific miRNAs. **A** Tissue distribution of miRNAs.The first is a Venn diagram of the number of miRNAs in the six tissues. The second one indicates the histogram of miRNAs contained in each tissue. The third one displays the number of miRNAs located in intersections of a specific size. In the third chart, 327 miRNAs are specific to one tissue, and 107 miRNAs are shared by two of the six lists. A miRNA is defined as expressed in a tissue if its reads count in this tissue is greater than 3. **B** MiRNA expression levels with different tissue shown as normalized read counts. **C** Common miRNAs show significantly higher expression than specific miRNAs. **D** Tissue-specific miRNAs expression. **E** MiRNAs tissue specificities in each group among the six species. Specific miRNAs gain high tissue specificities, while common miRNAs gain low tissue specificities. The tissue specificities were calculated based on each miRNA's fluctuations in expression levels

A total of 327 tissue-specific miRNAs were discovered (Fig. 5D), including three that were specifically expressed in muscle tissue, 11 in the intestine, 12 in the heart, 14 in the gills, 38 in the brain, and 249 in the hatching stage (Fig. 5A, Table S8). Grass carp-specific miRNAs were shown to be less abundant in grass carp than common miRNAs (Fig. 5B and C). Furthermore, grass carp-specific miRNAs are more tissue-specific than the common miRNAs (Fig. 5E). In conclusion, the expression levels of the majority of common miRNAs were plentiful in all tissues. Still, the expression levels of numerous grass carp-specific miRNAs were either low or restricted to specific tissues.

### Clustering generated the most abundant and essential grass carp-specific miRNAs

**Fig. 6:**
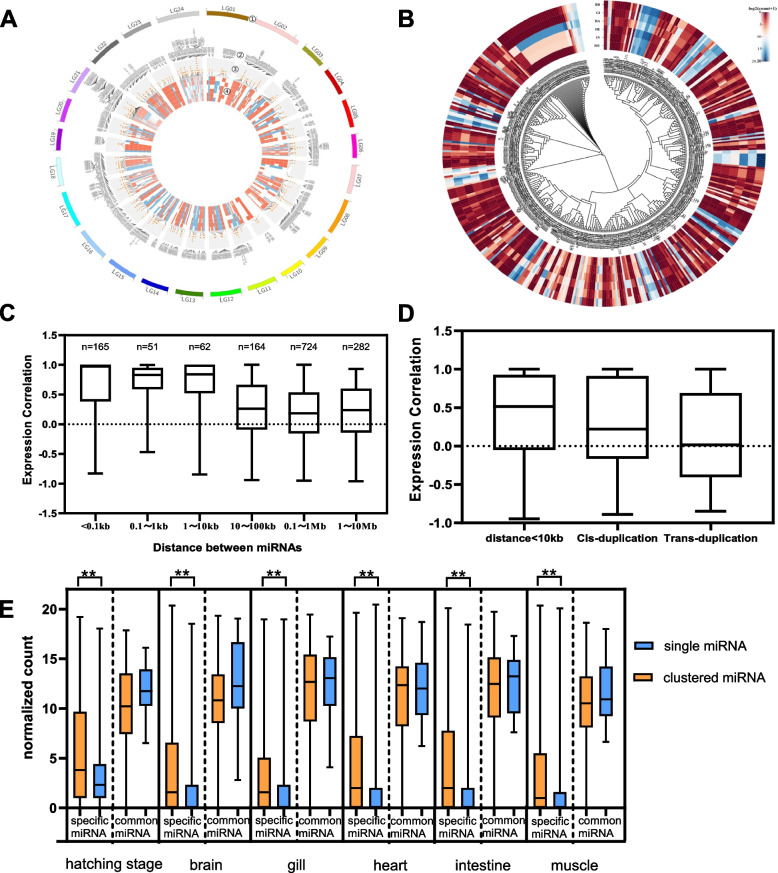
Expression patterns of pairs of neighboring miRNAs. **A** Clustered miRNAs show similar expression patterns. Tracks: 1. Linkage groups. 2. Name of clustered miRNAs. 3. miRNA loci, clustered miRNAs at adjacent positions, are displayed in a stacked style. 4. MiRNA expression level (log2 of normalized count add 1), from inside out: muscle, intestine, heart, hatching stage, gill, brain. **B** Expression profiles of miRNA genes. Tracks: 1. MiRNA expression level (log2 of normalized count add 1, in the same order as in plot A). 2. phylogeny of miRNA sequences. **C** Correlation between expression patterns of pairs of neighboring miRNAs. Expression correlations were calculated using data in Table S4. **D** Effects of distance and duplication, including remote (trans-) and local (cis-) miRNA duplication on miRNA expression. Only those miRNAs with well-defined duplicate types were considered (Table S5). **E** Specific miRNAs clustered with other miRNAs show significantly higher expression than specific single miRNAs. Comparisons between groups were analyzed by one-way analysis of variance (ANOVA). *p < 0.05 and **p < 0.01 indicated statistical significance

**Fig. 7:**
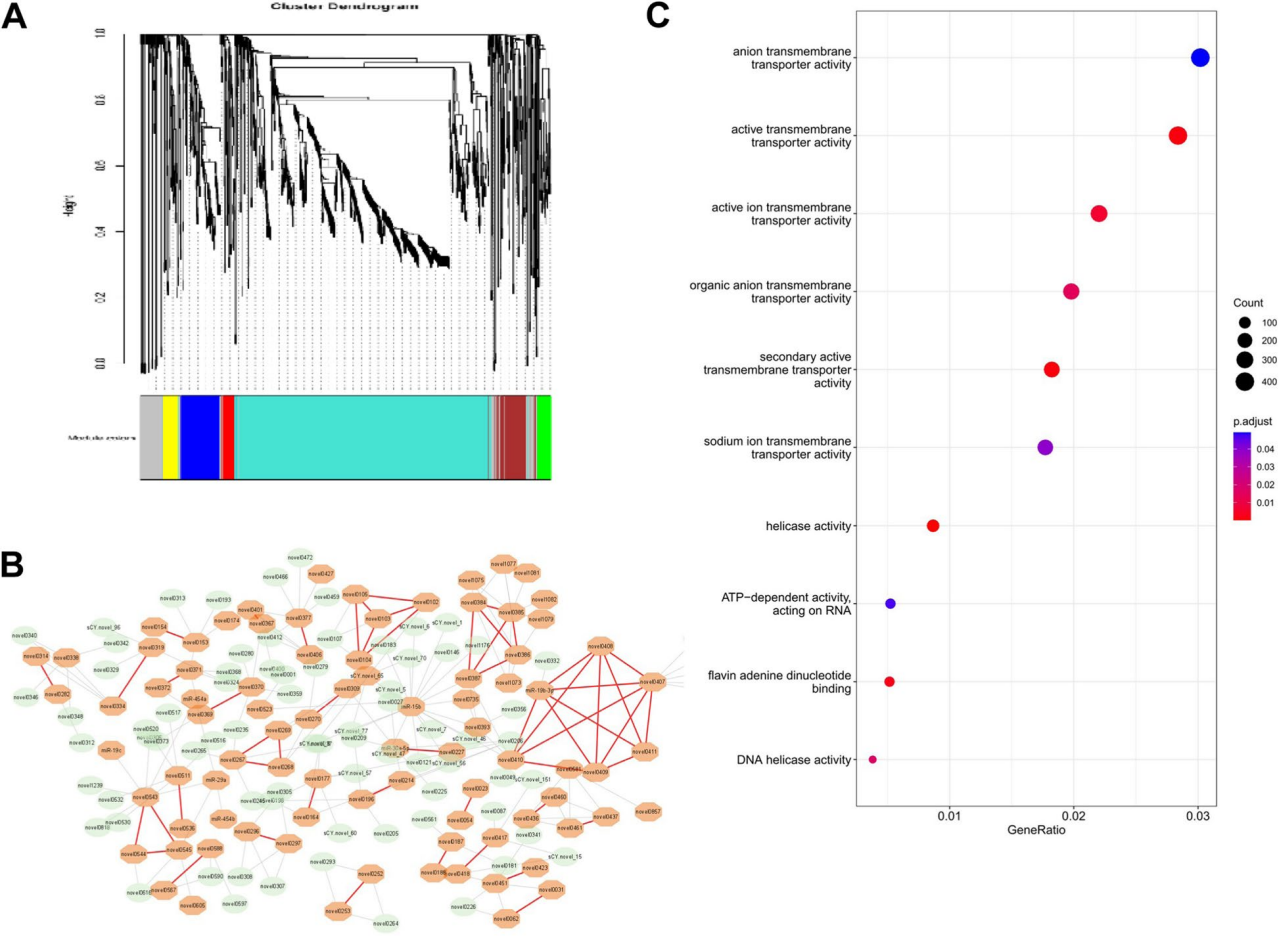
MiRNAs in the same clusters tend to be co-expressed. **A** Hierarchical cluster tree showing co-expression miRNA modules identified using WGCNA. Each leaf in the tree is one miRNA gene. Colors label modules corresponding to miRNAs. **B** A co-expression network of turquoise modules. The orange-filled octagons refer to clustered miRNAs. Red links represent the co-expressed miRNA cluster. **C** GO enrichment analysis of common miRNA

Following that, we investigate whether distance similarity (Fig. 6A) or sequence similarity (Fig. 6B) impact miRNA expression more. The expression of miRNAs with a distance range of less than 10 kb is highly correlated (Fig. 6C). This association was likewise significantly more robust than that of trans-duplications miRNA (Fig. 6D). In 103 of the 154 miRNA clusters that are expressed above thresholds in the analyzed libraries, all encoded miRNAs are categorized into the same expression modules (Fig. 7A, B, and Fig. S2). Clustered miRNAs can considerably enhance the connection between regulatory network nodes. Then, we compared the expression of single and clustered miRNAs. The presentation of clustered specific miRNAs was substantially higher than that of single specific miRNAs (Fig. 6E, Table S10).

GO enrichment data were found for common miRNA target genes. The GO enrichment analysis revealed that these common miRNAs were enriched in 10 molecular functions (MF), the most prominent of which are anion transmembrane transporter activity, active transmembrane transporter activity, and active ion transmembrane transporter activity (Fig. 7C,Table S11).

## Discussion

The initial evidence of miRNAs in teleosts was identified in 2003 in zebrafish (*Danio rerio*) [32]. Since miRNAs were discovered more than 20 years ago, fish miRNA research has made considerable strides. The research included the discovery of new miRNA in fish and an examination of the processes underlying the specific biological functions of miRNAs. In grass carp, 185 miRNAs associated with motile Aeromonad septicemia were found [33]. In rainbow trout, a total of 196 miRNA belonging to 124 families and 2,466 miRNA target genes was discovered [34]. Overexpression of miR-23a significantly suppression the glucagon signaling pathway [35]. However, there is still a lack of study on miRNA evolution in teleost. Current theories suggest that gene duplication, inversion, and variation are the key driving mechanisms for the emergence of a miRNA gene [36]. In human, some study discovered that expression level is a strong predictor of evolution rate. By studying three population samples and the sister species of *C. remanei*, Jovelin et al. demonstrated that natural selection continues to restrict the evolution of all sequence domains inside miRNA hairpins [37]. This work seeks to elucidate the aggregation pattern, evolution, and function of grass carp miRNAs.

In the present study, 1513 mature miRNAs were discovered, of which 1442 were specific to grass carp, demonstrating a high rate of gain for grass carp-specific miRNAs. Despite the high conservation of many miRNAs, many of the miRNAs are only found in one or two species that belong to a certain evolutionary branch. In contrast, others are present in at least three members of a particular evolutionary lineage [38]. For example, 453 (54.3%) of the 834 human miRNAs are either human-specific (130, 15.6%) or not conserved beyond primates (323, 38.7%) [39], whereas 430 (95.77%) of the 449 chicken miRNAs are avian-specific [40]. Only 56 of the 112 *Arabidopsis* miRNA genes are conserved in monocot rice, showing a significant percentage of unconserved miRNA genes [41]. Due to the limited number of miRNA genes, large rates of miRNA loss must have been balanced by high rates of gain [42]. Some miRNAs with poor conservation are deemed nonfunctional and evolutionarily transitory [43–46]. Due to the miRNAs can target numerous genes, some of which may be harmful to an organism. Consequently, certain miRNAs would undergo purifying selection, but others would perform essential functions and join the gene networks [47, 48]. Then, the expression levels of chosen miRNAs would grow, and they would acquire temporal and spatial specificity. Following this, specific miRNA sequences are likely to be preserved. This is the stage through which miRNAs evolve. Fish miRNA evolution through whole-genome duplication events emerged with a large number of miRNAs. In addition, an increase in the number of miRNA genes over time correlated with an increase in the complexity of postnatal organisms [4]. Teleost genome duplication (TGD) significantly contributed to the expansion of teleost miRNA gene repertoires. Similar to protein-coding genes, however, most miRNA gene losses occurred soon after the TGD and that the highest rate of miRNA gene loss occurred during the 85 My following the TGD [49]. Genomic context influenced retention rates, with miRNA clusters were retained more often than single miRNAs. The overall retention rate of clustered loci was significantly higher(+ 15.5%) than that of single miRNA loci in studies on zebrafish, *Oryzias latipes*, *Gasterosteus aculeatus*, and *Chaenocephalus aceratus* [50]. Finally, the major strands of miRNA genes underwent stronger purifying selection, especially in their seed and 30 complementary regions [51] that enables the emergence of novel targets and thus novel roles.

The process of “birth” and the development of new genes has garnered considerable interest among scientists. De novo formation, gene duplication, exon rearrangement, gene fusion/splitting, and horizontal gene transfer are all possible mechanisms. De novo formations are usually considered so unlikely that they are impossible in terms of earlier evolutionary processes [52]. In recent years, this view has been overturned by a wealth of evidence from various eukaryotic lineages. Available studies show that about 12% of new genes in the *Drosophila* genome originate de novo; about 60 de novo genes are known in the human genome, and at least 782 de novo genes are found in *Arabidopsis* [53]. Similar to protein-coding genes, the origin of young miRNA genes is usually achieved by duplication [5] or de novo formation [54, 55]. As hairpin structures are easy to form during RNA transcription, de novo formation is the most parsimonious mechanism for these miRNAs. A de novo formation gene may function by interacting with a pre-existing gene or provide an alternative function that causes the function of other genes to be suppressed and gradually lose its original function [56]. A recent study examining the evolution of miRNA genes in 12 *Drosophila* species estimated that only ~ 30% of gene gains within clusters could be explained by duplication events, with 46.1% of the new miRNAs in clusters originating from de novo formation [11]. Some miRNA genes are intergenic, and others reside within protein-coding genes, either on the same or the opposite DNA strand as the host gene and generally in introns and rarely in exons [10, 57, 58]; miRNAs tend to be clustered in introns or intergenic regions [8, 59]. A total of 171 miRNA clusters were identified in grass carp. The production pattern of 159 miRNAs within the clusters of miRNA genes is de novo. Our results are consistent with the previous results. Our findings imply that de novo creation accounts for a more significant proportion of the grass carp miRNA repertoire expansion.

New miRNAs, which always coincide with rapid evolution rates, are less conserved [60], have lowly expression levels [61], and appear to be species-specific [62], have been identified as a component of the overall process by which speciation occurs [63]. Strikingly, the evolution ratio for the single new miRNAs was significantly higher than that of the clustered, suggesting purifying selection might have driven the clustered new miRNAs to develop the function. The “functional coadaptation” hypothesis might provide light on the evolution and function of newly generated miRNA clusters [15]. Since miRNAs in the same clusters are usually co-transcribed temporally or spatially, the newly formed miRNAs might gradually develop functions to target genes that are related to the pre-existing miRNAs in the same cluster; or multiple de novo assembled new miRNAs in the same cluster interplay to regulate overlapping sets of target genes. The mouse miR-183–96-182 cluster monitors the entire signaling pathway by controlling three factors in the insulin signaling pathway [64]. This multi-target monitoring system appears more effective than the glucose-mediated extracellular insulin secretion pathway regulated by a single miR-375 [65]. Since those clustered miRNAs are primarily regulated under the same control, members within the same cluster could express and function cooperatively upon stimulation. The functions of individual miRNAs can be clarified by studying the processes of miRNA clusters, which are more complex and functionally efficient than individual miRNAs. Through the study of *Populus*-specific recently evolved miRNAs, new miRNAs have a greater chance of targeting over-retained copies of salicylic species, illustrating the potential role of *Populus*-specific recently evolved miRNAs in local adaptation and functional innovation [16]. Jovelin et al. hypothesize that new miRNAs may be responsible for regulatory variations amongst *Caenorhabditis* species [37]. These findings show that the study of species-specific miRNAs is essential for adaptive evolution.

Our findings indicated the evolutionary dynamic and genomic architecture of the grass carp miRNA repertoire. Grass carp miRNAs can exist in clusters and common miRNAs are more likely to be enriched in miRNA clusters. Grass carp-specific miRNAs evolve at a faster rate with a lower expression levels. It is claimed that the clustered arrangement of miRNAs has a remarkable effect on their origin in evolution and expression control. This work, which included comparative genomics and bioinformatics methodologies, was a worthwhile effort that yielded fresh insights into the origins and development of miRNA clusters in grass carp.

## Materials and methods

### Animals and embryos sampling

The experimental fish used in the experiment were from the Center of Grass Carp Breeding (Jiangsu, China). The tissue samples of grass carp juveniles (*n* = 15, average weight 40 g, average length 16 cm) were collected, including the brain, muscle, gill, intestine, and heart, and then these tissues were quickly frozen in liquid nitrogen and stored at -80 ℃. Embryos were obtained by artificial insemination spawning. According to the common carp model, the developmental phases of the embryo were split into seven stages and 24 periods, including the zygote, cleavage, blastula, gastrula, neurula, organogenesis, and hatching stages. In this work, we compared the differences in juvenile and embryonic development using hatching stages [66]. The embryo samples were immediately embedded in RNAlater (TIANGEN) and, after one night at 4 ℃, were stored at − 20 ℃ until RNA extraction. All treatments were performed with three biological replicates to ensure reproducibility of the data.

### RNA extraction, construction of a small RNA library, and deep sequencing

Total RNA extraction was performed using the mirVana miRNA Isolation Kit (Ambion) and the purity and integrity were evaluated with the Bioanalyzer 2100 system (Agilent Technologies, CA, USA). The analyses that followed employed samples with an RNA integrity number (RIN) ≥ 7. The TruSeq Small RNA Sample Prep Kits (Cat. No. RS-200–0012, Illumina, USA) were used in accordance with the manufacturer's instructions to prepare 1 μg of total RNA for the creation of the small RNA library. Briefly, total RNA was ligated to adapters at each end. Then the adapter-ligated RNA was reverse transcribed to cDNA and performed PCR amplification. DNA fragments corresponding to sizes of 140–160 bp were recovered. DNA High Sensitivity Chips were used to evaluate library quality on the Agilent Bioanalyzer 2100 system. Small RNA transcriptome sequencing were conducted by OE Biotech Co., Ltd. (Shanghai, China) with the Illumina HiSeq X Ten platform.

### Annotation of known sRNAs and discovery of novel miRNAs

Initial reads were processed by removing poor quality reads, 5' adapter pollution reads, reads without 3' adapter, reads without insert fragment, reads containing poly(A) stretches, and reads less than 18 nt or longer than 30 nt. The qualified reads that passed the filters above were then aligned to grass carp genome, known miRNAs, repeat sequences, exon and intron sequences using Bowtie version 1.3.1 [67] with the parameters -v 0 -a -best -strata. These RNAs were aligned and then annotated against the Rfam database (http://rfam.xfam.org/) [68] allowing for two mismatches. To make every unique small RNA mapped to only one annotation, we followed the following priority rule: known miRNA > ncRNA > repeat > exon > intron. After that, unannotated reads were analyzed by miRDeep2-v0.1.3 [69] to predict novel miRNAs.

### Expression of miRNAs and target prediction

The expression levels of miRNAs within each library were normalized to give the expression in transcripts per million mapped reads (TPM). Target prediction was performed by integrating three miRNA target prediction software packages, including miRanda v3.3a, RNAhybrid v2.1.2, and targetscan_70 [70]. We extracted mRNA sequences from transcriptome data and used them as potential candidates in miRNA target prediction. MiRanda parameter was: energy threshold, − 10.0. Also, miRanda strict option was used, requiring exact alignment in the seed region (miRNA positions 2–8). RNAhybrid energy threshold was set at − 20.0.

### Phylogenetic distribution of miRNA families

The genome sequences of *Ciona intestinalis*, *Branchiostoma floridae*, *Salmo salar*, *Danio rerio*, *Cyprinus carpio*, *Xenopus tropicalis*, *Anolis carolinensis*, *Gallus gallus*, *Ovis aries*, *Bos taurus*, and *Petromyzon marinus* were downloaded from Ensembl (https://asia.ensembl.org/index.html). The miRNA sequences of all the other species except *C. idella* were downloaded from miRbase (https://www.mirbase.org/). The birth, death, and age of miRNA families were estimated based on a phylogenetic tree generated by RAxML(-m PROTGAMMAJTT -f a -# 100) [71] analysis from single-copy proteins*.* The birth, death, and age of miRNA families and the ancestral gene contents were assessed using the COUNT software [72] following the parsimony rule based on the miRNA families of each species. Branch lengths reflect evolutionary divergence times in million years (MY) inferred from timeTree (http://www.timetree.org/).

### Origin of new miRNAs in miRNA clusters

A miRNA cluster was defined if neighboring miRNAs were located within 10 kb. Because ancient events of miRNA genesis might be challenging to determine, inherited miRNAs were not examined. We also omitted the initial miRNA in a cluster since it appeared as a single miRNA incapable of forming a cluster on its own. To identify clustered miRNA origin, the mature miRNAs were compared with each other by an all-against-all blast. Then, they were clustered according to sequence similarity, requiring alignment ≧ 15 nt, which covers the miRNA seed region ( position 2–8 nt) and mismatch ≦ 2. Consider clustered miRNAs that comply with the following requirements evolved by duplication. There are two types of miRNA duplication: cis-duplication between adjacent locations and trans-duplication between different chromosomal locations. MiRNAs that don't fit the criteria above are thought to evolve by de novo formation.

### MiRNA evolution rates

Select miRNA flanking sequences with the same length as the miRNA precursor. Then the alignments for precursors and flanking sequences were constructed using T-coffee (https://www.ebi.ac.uk/Tools/msa/tcoffee/) with the default parameters. From these alignments, we calculated the sequence divergence between the miRNA precursors and their homologous sequences using KaKs_calculator 3.0 software [73], marked as K_p_. We then calculated the sequence divergence between the flanking sequences and their homologous sequences, marked as K_f_, in the same way. The miRNA evolutionary rate was calculated as K = K_p_ / K_f_.

### Collinearity analysis of *C. idella* miRNA genes

Collinear blocks harboring orthologous miRNAs between *Ctenopharyngodon idella* and *Danio rerio* were determined using MCScanX [74]. The miRNAs in *C. idella* were used to search against the *D. rerio* genome sequence using BLASTN with an E-value of 0.01. The miRNA pairs and the corresponding miRNA position in *C. idella* were used as input when running MCScanX. The miRNA pairs were considered interspecific orthologs if the miRNA genes were in the same flanking syntenic region between *C. idella* and *D. rerio*. The protein sequences of *D. rerio* were downloaded from Ensembl (https://asia.ensembl.org/index.html).

### Tissue specificity measurement

To measure the tissue specificity, the tissue specificity score [75]was computed thus: Let a_ij_ be the average expression of gene *i* in tissue *j*. Then the tissue specificity of gene *i* is defined by:

$${\mathrm{T}}_{\mathrm{i}}=\frac{1}{\mathrm{n}-1}{\sum }_{j=1}^{n}\left(1-\frac{{a}_{ij}}{{max}_{j}({a}_{ij})}\right)$$

where *n* is the number of tissues. Thus, if a gene is expressed in only one tissue, the score is 1; if the expression of a gene is the same in all tissues, the score is 0.

### Gene network construction and visualization

Co-expression networks were constructed using the WGCNA package in R [76]. Normalized expression data were used for the WGCNA unsigned co-expression network analysis. All parameters were defined except “soft_power = 18, min_module_size = 30”. The networks were visualized using Cytoscape _v.3.9.1.

## Supplementary Information

**Additional file 1: Fig. S1.** Detailed information of miRNAs collinear blocks. Collinear blocks between the two species are shown in blue.

**Additional file 2: Fig. S2.** Co-expression networks of modules. (A) A co-expression network of the blue module. (B) A co-expression network of the green module. (C) A co-expression network of the grey module. (D) A co-expression network of the red module. (E) A co-expression network of the brown module. (F) A co-expression network of the yellow module. The orange-filled octagons refer to clustered miRNAs. Red links represent the co-expressed miRNA cluster.

**Additional file 3: Table S1.** miRNA location in the grass carp genome. **Table S2.** The miRNA family of twelve species analyzed. **Table S3.** miRNA clusters in the grass carp genome. **Table S4.** Expression correlation between miRNA duplication of grass crap. **Table S5.** MiRNA evolution rate. **Table S6.** The colinearity blocks of protein between grass carp and zebrafish. **Table S7.** The colinearity blocks of miRNA between grass carp and zebrafish. **Table S8.** Tissue-specific miRNAs in the grass carp. **Table S9.** Expression correlation between neighbouring miRNAs of grass carp. **Table S10.** Expression level of grass carp miRNAs with different genomic context. **Table S11.** The predicted common miRNA/target pairs.

## Data Availability

All the data supporting our findings are contained within the manuscript. Raw Illumina sequences have been deposited into the NCBI’s Sequence Read Archive (SRA) database with accession numbers SRP398370.

## Abbreviations

miRNA: MicroRNAs
MID: Maximum inter-miRNA distance
GO: Gene Ontology
TPM: Transcripts per million
WGCNA: Weighted co-expression network analysis
