# Supplementary material for: Clustering pattern and evolution characteristic of microRNAs in grass carp (Ctenopharyngodon idella)
Source: BMC Genomics. 2023 Feb 13;24:73. doi: 10.1186/s12864-023-09159-x (PMC9926789; doi:10.1186/s12864-023-09159-x)
Supplement: Supplementary file 1 — Additional file 1: Fig. S1. Detailed information of miRNAs collinear blocks. Collinear blocks between the two species are shown in blue. [file 12864_2023_9159_MOESM1_ESM.pdf]

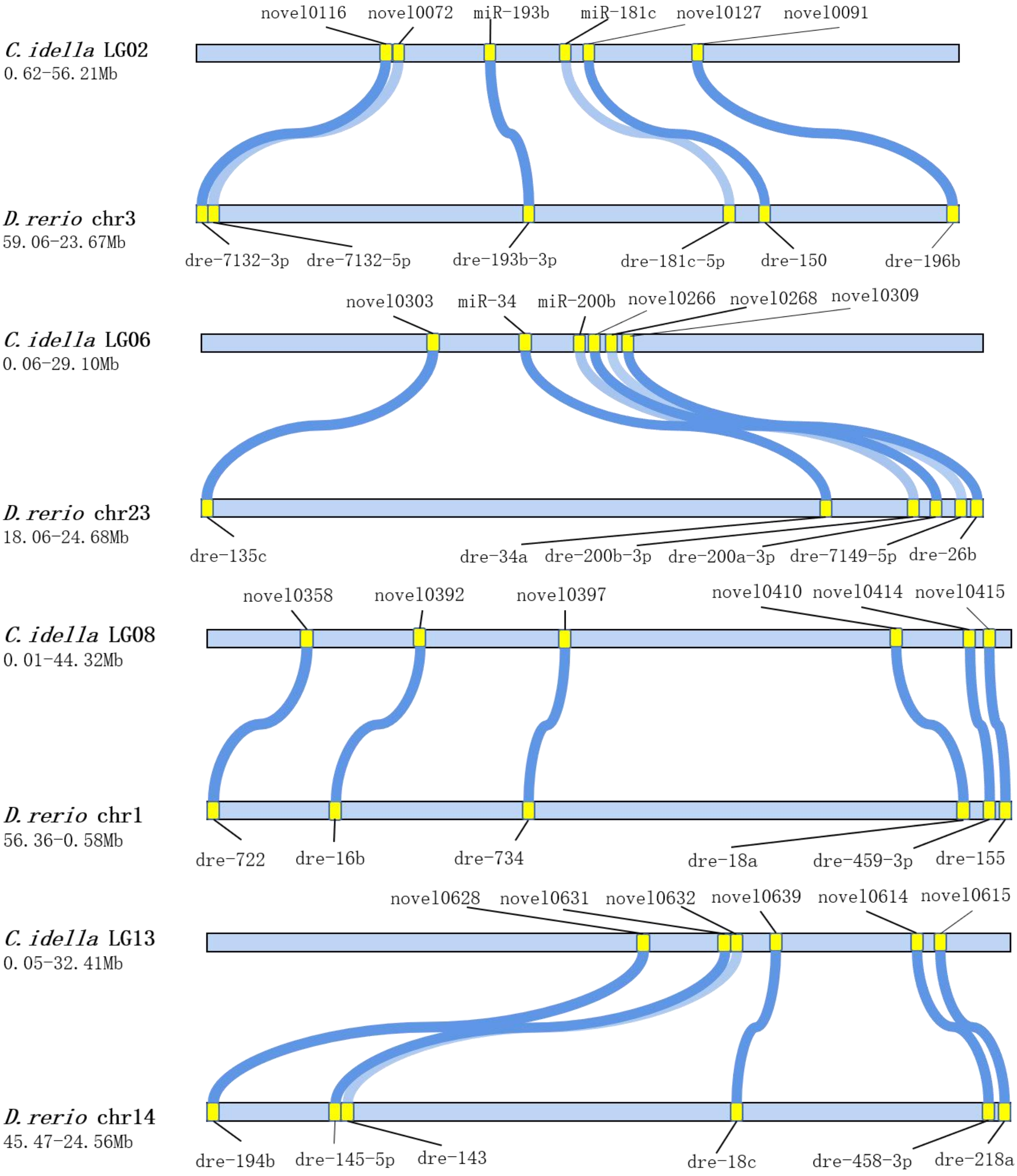

Fig. S1 Detailed information of miRNAs collinear blocks.
Collinear blocks between the two species are shown in blue.
