# Supplementary material for: Clustering pattern and evolution characteristic of microRNAs in grass carp (Ctenopharyngodon idella)
Source: BMC Genomics. 2023 Feb 13;24:73. doi: 10.1186/s12864-023-09159-x (PMC9926789; doi:10.1186/s12864-023-09159-x)
Supplement: Supplementary file 2 — Additional file 2: Fig. S2. Co-expression networks of modules. (A) A co-expression network of the blue module. (B) A co-expression network of the green module. (C) A co-expression network of the grey module. (D) A co-expression network of the red module. (E) A co-expression network of the brown module. (F) A co-expression network of the yellow module. The orange-filled octagons refer to clustered miRNAs. Red links represent the co-expressed miRNA cluster. [file 12864_2023_9159_MOESM2_ESM.pdf]

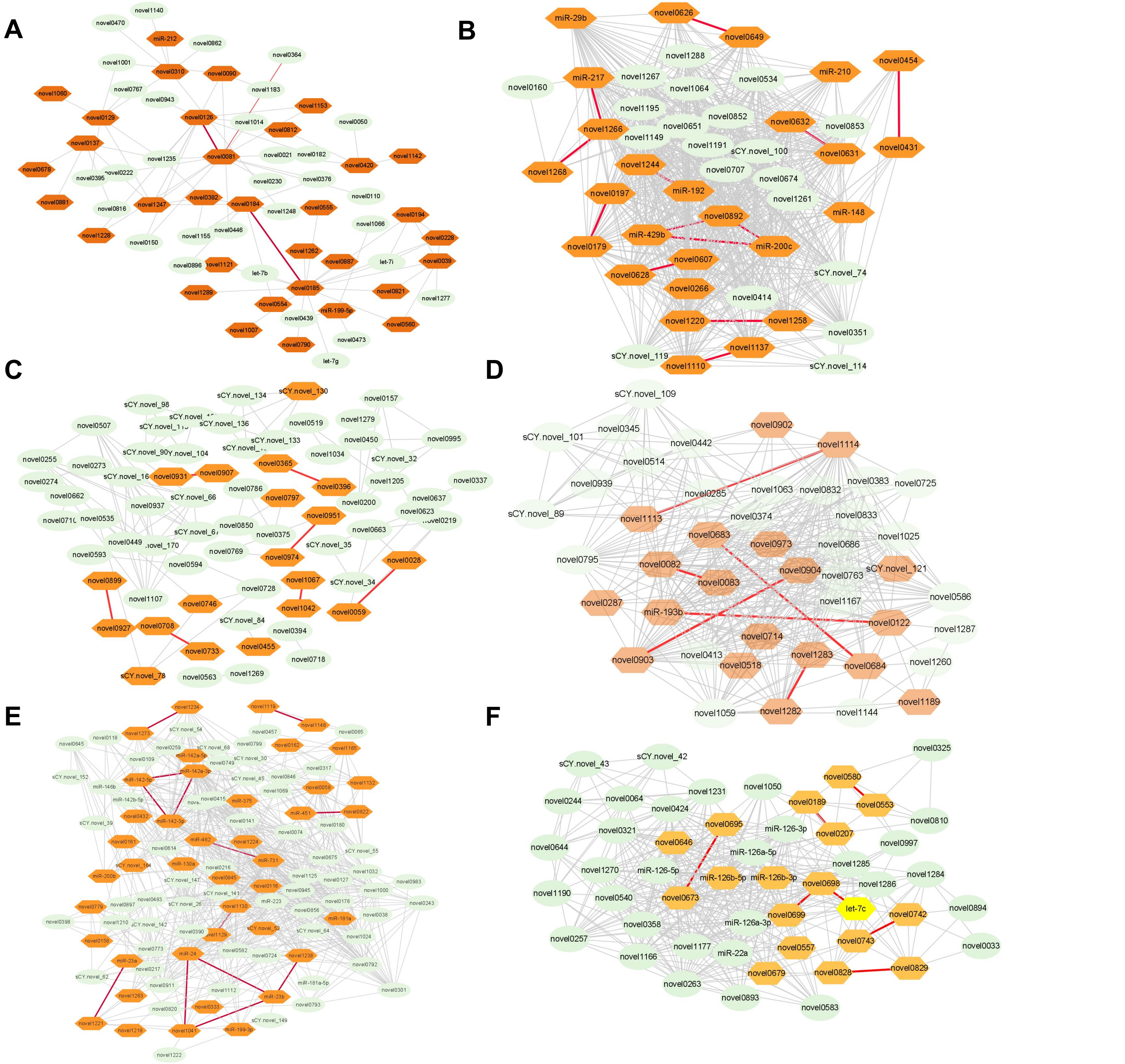

Fig. S2 Co-expression networks of modules.

(A) A co-expression network of the blue module. (B) A co-expression network of the green module. (C) A co-expression network of the grey module. (D) A co-expression network of the red module. (E) A co-expression network of the brown module. (F) A co-expression network of the yellow module. The orange-filled octagons refer to clustered miRNAs. Red links represent the co-expressed miRNA cluster.
